# Statin Out-of-Pocket Expenditures Under Private Insurance vs Medicaid After 2016 USPSTF

**DOI:** 10.1001/jamanetworkopen.2025.37041

**Published:** 2025-10-10

**Authors:** Ben Mosier, Lynn M. Hua

**Affiliations:** 1 Department of Economics, Andrew Young School of Policy Studies, Georgia State University, Atlanta; 2 Greenberg School of Risk Science, Robinson College of Business, Georgia State University, Atlanta

## Abstract

This economic evaluation compares out-of-pocket costs for statins among privately insured vs Medicaid enrollees before and after the 2016 US Preventive Services Task Force (USPSTF) grade B recommendation.

## Introduction

US out-of-pocket statin expenditures were $3.9 billion in 2012-2013.^1^ In 2016, the US Preventive Services Task Force (USPSTF) issued a B-grade recommendation, indicating moderate certainty of a moderate-to-high substantial benefit, for statins to adults aged 40-75 years without cardiovascular disease (CVD) but 1 or more CVD risk factors and a 10% or greater 10-year CVD event risk.^2^ The Affordable Care Act (ACA) mandates that nongrandfathered private insurance plans cover USPSTF A- or B-graded preventive services with 0 cost-sharing.^3^ This study documented trends in statin expenditures for privately insured vs Medicaid enrollees before and after the 2016 recommendation. Legal challenges to the ACA mandate, like *Kennedy v Braidwood*, underscore the policy significance of these findings.^4^

## Methods

The Georgia State University Institutional Review Board approved this economic evaluation and waived informed consent because the study used deidentified, publicly available data. Reporting followed the CHEERS reporting guideline. We obtained prescription expenditure data from the nationally representative Medical Expenditure Panel Survey (MEPS), where patients can be repeatedly sampled for up to 2 years, from 2013-2022. The sample included adults aged 40-64 years with private health insurance or Medicaid with a statin prescription fill during the calendar year and no CVD history. Outcome variables included a binary indicator of 0 out-of-pocket payments for statins and estimated out-of-pocket cost for an annual statin supply. This cost was calculated by multiplying each patient’s cost per dose from their first filled statin in each year (including those with no out-of-pocket costs) by 365 to account for variation in plan benefit designs. Estimates reflect cost differences for patients who would have remained in the same insurance plan without reaching their deductible. The final sample included 9522 and 6227 observations for 0 cost-sharing and out-of-pocket cost outcomes, respectively.

A difference-in-differences event-study regression model compared changes in outcomes relative to the 2016 USPSTF recommendation across privately insured and Medicaid enrollees. We included controls for heart disease risk factors, demographic characteristics, and year-by-drug formulation-by–generic status fixed effects. Results were estimated using MEPS survey weights. Sensitivity tests varied modeling assumptions, inclusion of all prescription fills, and addition of other public insurance in the control group (eMethods in Supplement 1). Analyses used Stata version 17 (StataCorp), with 2-sided *P* values evaluated at the .05 level and 95% CIs.

## Results

Our sample included 9564 observations of patient-years (mean age, 55.2 years [95% CI, 55.1-55.4 years]; 55.5% [95% CI, 54.4%-56.7%] male; 10.0% [95% CI, 9.3%-10.7%] Black, 11.8% [95% CI, 11.1%-12.6%] Hispanic, and 80.7% [95% CI, 79.6%-81.7%] White). The Table presents mean demographics over year ranges. In 2016, 41.7% (95% CI, 33.0%-51.0%) of Medicaid and 8.2% (95% CI, 5.7%-10.8%) of privately insured statin users paid no out-of-pocket costs for statins. By 2022, these percentages increased to 55.5% (95% CI, 45.3%-65.7%) and 46.4% (95% CI, 41.2%-51.5%), respectively.

**Table.** Study Sample Characteristics^a^

Characteristic	Participants, mean (SD), %		Difference, % (95% CI)	*P* value
	Medicaid	Private insurance		
Age, mean (SD), y	54.7 (6.5)	55.4 (6.4)	0.7 (0.3 to 1.1)	.001
Sex				
Male	41.9 (49.4)	58.3 (49.3)	16.3 (13.6 to 19.1)	<.001
Female	58.1 (49.4)	41.7 (49.3)	−16.3 (−19.1 to −13.6)	<.001
Race and ethnicity				
Black	17.8 (38.2)	8.4 (27.8)	−9.3 (−11.3 to −7.4)	<.001
Hispanic	21.4 (41.0)	9.9 (29.9)	−11.5 (−13.6 to −9.3)	<.001
White	70.0 (45.9)	82.8 (37.7)	12.9 (10.3 to 15.5)	<.001
Other^b^	12.3 (32.8)	8.8 (28.3)	−3.5 (−5.5 to −1.6)	<.001
High cholesterol	89.1 (31.2)	90.8 (29.0)	1.7 (0.0 to 3.3)	.05
High blood pressure	69.0 (46.3)	58.9 (49.2)	−10.1 (−12.9 to −7.3)	<.001
Diabetes	45.7 (49.8)	27.1 (44.4)	−18.6 (−21.4 to −15.7)	<.001
Household income, $	31 476.22 (34 847.13)	113 351.19 (77 202.12)	81 820.59 (78 896.18 to 84 745.01)	<.001
Bachelor’s degree or higher	14.3 (35.0)	48.6 (50.0)	34.3 (32.0 to 36.7)	<.001
Zero OOP cost				
2013-2016	39.0 (48.8)	6.8 (25.2)	−32.2 (−36.9 to −27.5)	<.001
2018-2022	53.7 (49.9)	47.8 (50.0)	−5.9 (−10.3 to −1.6)	.007
Estimated annual OOP cost, $				
2013-2016	31.44 (111.57)	125.26 (298.06)	93.82 (71.71 to 115.93)	<.001
2018-2022	12.70 (44.57)	45.04 (86.50)	32.34 (27.15 to 37.52)	<.001

Abbreviation: OOP, out of pocket.

^a^ All statistics were calculated using Medical Expenditure Panel Survey (MEPS) weights.

^b^ Participant race and ethnicity were self-reported via the MEPS survey instrument across 2 separate survey questions. Race was determined via the RACEA variable. Options for race were Alaskan Native or American Indian, Asian, Black, White, or multiple races. In the analysis, all race groups other than Black and White were assigned to other. The ethnicity question, variable HISPYN in the MEPS survey, was a yes or no question whether the respondent considered themselves Hispanic or Latino. Race and ethnicity were queried in separate survey questions.

Annual differences in 0 out-of-pocket statin cost between privately insured and Medicaid enrollees showed increases for the privately insured ranging from 20.5 percentage points (95% CI, 9.0 to 32.0 percentage points; *P* < .001) to 42.3 percentage points (95% CI, 30.1 to 54.6 percentage points; *P* < .001) (Figure, A). Annual differences in estimated annual out-of-pocket costs between privately insured and Medicaid enrollees ranged from a nonsignificant $11.74 (95% CI, –$4.89 to $28.36; *P* = .17) to $32.14 (95% CI, $16.25 to $48.04; *P* < .001) (Figure, B).

**Figure.**
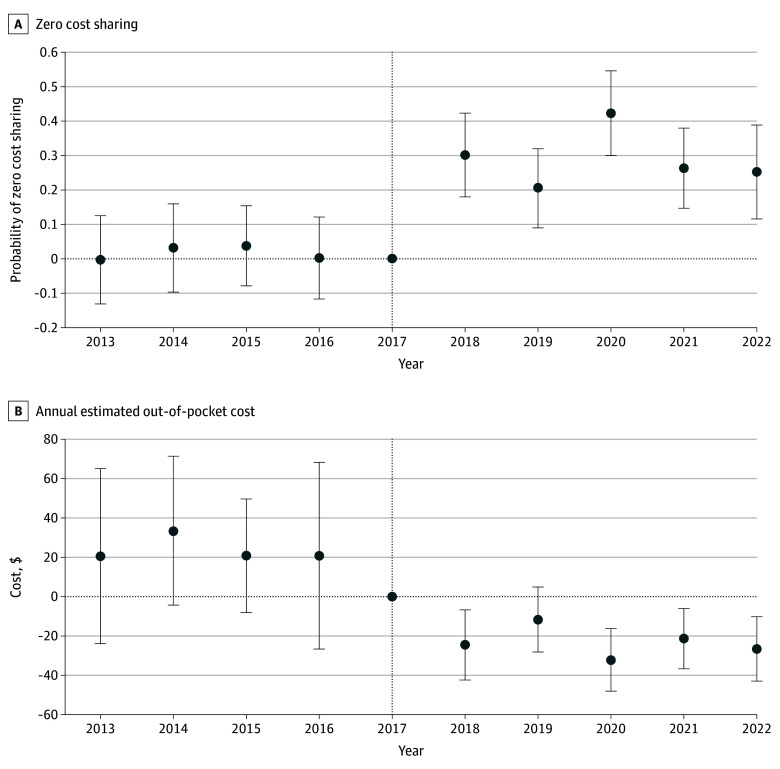
Event-Study Difference-in-Differences Estimates of Annual Effects on Zero Cost-Sharing and Annual Out-of-Pocket Costs Among Patients With Private Insurance vs Medicaid Who Were Prescribed Statins Difference-in-differences event study estimates are presented for 0 cost-sharing (A) and annual estimated out-of-pocket cost (B) between privately insured and Medicaid enrollees from 2013 to 2022. Whiskers indicate 95% CIs. The vertical line at 2017 denotes that 2017 is the reference year in the event study analysis.

## Discussion

This economic evaluation found that after the USPSTF recommendation, privately insured individuals experienced notable increases in 0 cost-sharing and reductions in out-of-pocket cost compared with Medicaid enrollees. Given the large scale of statin expenditures, a potential reversal of these cost reductions through future legal challenges may be associated with substantial increases in patient out-of-pocket spending.

Despite these cost reductions, a substantial portion of statin users incurred out-of-pocket costs, although they met criteria for 0 cost-sharing. This affirms that frictions remain between insurers, health care institutions, and patients despite the ACA mandate.^5^ Our study’s main limitation is that we could not observe detailed plan benefit design information. Additionally, MEPS is known to underestimate spending, and health characteristics are subject to reporting bias.^6^ Whether this incomplete compliance can be explained by restrictions on brand-name drugs or administrative frictions, such as prior authorization and preventive service coding, remains a key area for future research.

## Notes

Supplement 1.
eMethods.

Supplement 2.
Data Sharing Statement
