# Supplementary material for: Statin Out-of-Pocket Expenditures Under Private Insurance vs Medicaid After 2016 USPSTF
Source: JAMA Netw Open. 2025 Oct 10;8(10):e2537041. doi: 10.1001/jamanetworkopen.2025.37041 (PMC12514622; doi:10.1001/jamanetworkopen.2025.37041)
Supplement: Supplement 1. — eMethods. [file jamanetwopen-e2537041-s001.pdf]

Supplemental Online Content

Mosier B, Hua LM. Statin out-of-pocket expenditures under private insurance vs Medicaid after 2016 USPSTF. *JAMA Netw Open*. 2025;8(10):e2537041.
doi:10.1001/jamanetworkopen.2025.37041

eMethods.

This supplemental material has been provided by the authors to give readers additional information about their work.

© 2025 Mosier B et al. *JAMA Network Open*.

eMethods.

Statin Drug Sample:

IPUMS MEPS variable **RXNAME** was used to construct unique statin/generic binary indicator variables to control for price differences. Per IPUMS MEPS, all medication-round fills, except those determined to be a confidentiality risk, **RXNAME** reports the unedited pharmacy-provided name of the prescribed medicine.

Final list of statin drugs, generic versions, and approval years:

Mevacor/Altoprev (lovastatin, generic approved 2001)
Zocor (simvastatin, 2006)
Pravachol (pravastatin, 2006)
Lipitor (atorvastatin, 2011)
Lescol (fluvastatin, 2012)
Crestor (rosuvastatin, 2016)
Livalo (pitavastatin, 2020)

Variables Used to Calculate Outcomes (definitions per IPUMS MEPS):

For all medication-round fills, **RXFEXPSELF** captures the amount paid for the prescribed medicine fill by the person and the person's family.

For all medication-round fills, **RXDAYSUP** reports the number of days supplied for the medication fill.

Sample Restriction and Control Variables (definitions per IPUMS MEPS):

To limit the sample to individuals without prior cardiovascular disease (CVD), we excluded respondents with any relevant ICD-coded diagnoses or self-reported heart conditions. Specifically, we excluded those with ICD-9-CM codes 410–412 (ischemic heart disease), 414 (chronic ischemic heart disease), 424–429 (other heart diseases), V12 and V15 (personal history of CVD), and V45 (cardiac device/aftercare); and ICD-10-CM codes I20–I25 (ischemic heart disease), I42–I51 (other heart diseases), Z86 (personal history of disease), and Z95 (presence of cardiac device). We also excluded individuals who answered affirmatively to any of the following IPUMS MEPS condition variables: CHEARTDIEV (coronary heart disease), HEARTATTEV (heart attack), HEARTCONEV (other heart disease), or STROKEV (stroke or TIA).

ICD9CODE reports the ICD-9-CM diagnosis code for all current conditions. To preserve confidentiality, AHRQ offers a version of the ICD-9-CM codes where the fully specified ICD-

© 2025 Mosier B et al. *JAMA Network Open*.

9-CM codes have been collapsed to 3-digit code categories. Beginning in 2016, conditions are coded to ICD-10-CM codes (ICD10CODE), and not ICD-9-CM codes in MEPS data.

ICD10CODE reports the ICD-10-CM code for all current conditions associated with a medical event or prescribed medicine during the year. To preserve confidentiality, AHRQ collapsed the fully specified ICD-10-CM codes into 3-digit diagnosis code categories.

CHOLHIGHEV identifies persons over age 17 who have been diagnosed with high cholesterol. Respondents were asked if they have ever been told "by a doctor or other health professional that (PERSON) had high cholesterol?"

HYPERTENEV identifies persons over age 17 who have been diagnosed with hypertension. Respondents were asked if they have ever been told "by a doctor or other health professional that (PERSON) had hypertension, also called high blood pressure?"

DIABETICEV identifies persons over age 17 who have been diagnosed with diabetes, other than during pregnancy. Respondents were asked if, other than during pregnancy, they have ever been told "by a doctor or other health professional that (PERSON) had diabetes or sugar diabetes?"

CHEARTDIEV identifies persons over age 17 who have been diagnosed with coronary heart disease. Respondents were asked if they have ever been told "by a doctor or other health professional that (PERSON) had coronary heart disease?"

HEARTATTEV identifies persons over age 17 who have had a heart attack. Respondents were asked if they have ever been told "by a doctor or other health professional that (PERSON) had a heart attack, also called myocardial infarction or MI?"

HEARTCONEV identifies persons over age 17 who have been diagnosed with a heart condition or heart disease other than coronary heart disease, angina, or heart attack. Respondents were asked if they have ever been told "by a doctor or other health professional that (PERSON) had any other kind of heart condition or heart disease, other than coronary heart disease, angina, or heart attack?"

STROKEV identifies persons over age 17 who have had a stroke. Respondents were asked if they have ever been told "by a doctor or other health professional that (PERSON) had a stroke or TIA? A TIA is a transient ischemic attack which is sometimes referred to as a ministroke."

AGE (definition per IPUMS MEPS): AGE reports the individual's exact age, calculated from date of birth, as of the last day (12/31) of the survey year.

RACEA (definition per IPUMS MEPS): RACEA reports the race of the respondent. If not ascertained, the race and/or ethnicity were assigned based on relationship to other

© 2025 Mosier B et al. *JAMA Network Open*.

members of the DU using a priority ordering that gave precedence to blood relatives in the immediate family.

HISPYN (definition per IPUMS MEPS): For all persons, HISPYN indicates if the respondent considers her/himself Hispanic or Latino.

EDUC reports the highest level of schooling an individual had completed, in terms of completed grades for persons with less than a high school degree, and in terms of degrees attained for high school graduates and those with higher education. EDUC is available for all survey participants age 5 and older at the time of their first MEPS interview. It is an IPUMS MEPS constructed variable.

Robustness Tests:

F-tests for the joint significance of the pre-treatment coefficients in the event study regressions were conducted for each outcome: zero cost-sharing and estimated out-of-pocket cost per year. For both outcomes, we failed to reject the null hypothesis that all pre-treatment coefficients are equal to zero (F = 0.44, *P* = 0.78 for zero cost-sharing; F = 0.85, *P* = 0.49 for out-of-pocket cost).

A sensitivity analysis was performed that added publicly insured statin users to the sample who were insured by sources other than Medicaid. This increased sample size from 13,668 to 15,696 patient-years for the no-cost sharing outcome, and from 9,100 to 10,659 for estimated out-of-pocket costs. The results are consistent with our main model specification.

A placebo test was also conducted where the binary treatment variable was assigned to 2015, two years prior to the policy change. Results demonstrated the statistically significant differences between the treatment and control group are isolated to years 2018 through 2022, after the policy change.

© 2025 Mosier B et al. *JAMA Network Open*.
