# Supplementary material for: Statin Out-of-Pocket Expenditures Under Private Insurance vs Medicaid After 2016 USPSTF
Source: JAMA Netw Open. 2025 Oct 10;8(10):e2537041. doi: 10.1001/jamanetworkopen.2025.37041 (PMC12514622; doi:10.1001/jamanetworkopen.2025.37041)
Supplement: Supplement 2. — Data Sharing Statement [file jamanetwopen-e2537041-s002.pdf]

Data Sharing Statement

Mosier. Statin Out-of-Pocket Expenditures Under Private Insurance vs Medicaid After 2016 USPSTF. *JAMA Netw Open*. Published online October 10, 2025. doi:10.1001/jamanetworkopen.2025.37041

Data
Data available: Yes
Data types: Deidentified participant data
How to access data: Data and code will be available upon request at rmosier2@student.gsu.edu
When available: With publication

Supporting Documents
Document types: Statistical/analytic code
How to access documents: rmosier2@student.gsu.edu
When available: With publication

Additional Information
Who can access the data: Anyone requesting the data
Types of analyses: Any purpose
Mechanisms of data availability: Without investigator support
